# Multivariate Genome-Wide Association Study of Concentrations of Seven Elements in Seeds Reveals Four New Loci in Russian Wheat Lines

**DOI:** 10.3390/plants12173019

**Published:** 2023-08-22

**Authors:** Nadezhda A. Potapova, Anna N. Timoshchuk, Evgeny S. Tiys, Natalia A. Vinichenko, Irina N. Leonova, Elena A. Salina, Yakov A. Tsepilov

**Affiliations:** 1 Kurchatov Genomics Center, Institute of Cytology and Genetics, Siberian Branch of the Russian Academy of Sciences, 630090 Novosibirsk, Russia; 2 Institute for Information Transmission Problems (Kharkevich Institute), Russian Academy of Sciences, 127051 Moscow, Russia; 3 Institute of Cytology and Genetics, Siberian Branch of the Russian Academy of Sciences, 630090 Novosibirsk, Russia; 4 MSU Institute for Artificial Intelligence, Lomonosov Moscow State University, 119991 Moscow, Russia

**Keywords:** wheat, *Triticum aestivum*, microelement, macroelement, GWAS, multivariate analysis

## Abstract

Wheat is a cereal grain that plays an important role in the world’s food industry. The identification of the loci that change the concentration of elements in wheat seeds is an important challenge nowadays especially for genomic selection and breeding of novel varieties. In this study, we performed a multivariate genome-wide association study (GWAS) of the seven traits—concentrations of Zn, Mg, Mn, Ca, Cu, Fe, and K in grain—of the Russian collection of common wheat *Triticum aestivum* (N = 149 measured in two years in two different fields). We replicated one known locus associated with the concentration of Zn (IAAV1375). We identified four novel loci—BS00022069_51 (associated with concentrations of Ca and K), RFL_Contig6053_3082 (associated with concentrations of Fe and Mn), Kukri_rep_c70864_329 (associated with concentrations of all elements), and IAAV8416 (associated with concentrations of Fe and Mn)—three of them were located near the genes TraesCS6A02G375400, TraesCS7A02G094800, and TraesCS5B02G325400. Our result adds novel information on the loci involved in wheat grain element contents and may be further used in genomic selection.

## 1. Introduction

Wheat is a cereal grain that plays an important role in the world’s food industry. Wheat lines have been studied using many genomic approaches, such as genome sequencing and resequencing [1,2,3]), transcriptome analysis [4,5,6], and SNP arrays [7,8,9], that have given one an opportunity to investigate many wheat traits, such as element concentrations, immune response, and drought resistance. 

One of the important components of wheat seeds are micronutrients, such as calcium, zinc, and magnesium. Their concentrations are of special interest for genomic selection studies due to their role in grain food value. Micronutrient deficiency, also known as hidden hunger, is a result of food intake with low concentrations of micronutrients and vitamins [10]. It may lead to different diseases and even death. For that reason, an ability to increase concentrations of different micronutrients, including elements widely used in the food industry in wheat and its products, is an important option to affect the current situation with micronutrient deficiency in the world. Many attempts were made to uncover genomic loci affecting lower or higher concentrations of certain elements [11,12,13,14,15,16,17,18], such as Zn, Fe, Cu, Mn, and P. 

The identification of the loci-changing element concentrations as well as genomic selection for concentrations is an important goal in agronomy nowadays. In this respect, special attention is to be paid to local varieties, which are adapted to particular climatic and geographical conditions of the place where they are grown. Russia ranks first in wheat exports in the world (FAOSTAT); therefore, studying Russian wheat lines and searching for the loci important for wheat quality leads to improving the food situation in the world. Until now, there has been only one study on spring Russian varieties [19] where spring wheat germplasm and concentrations of macro- and microelements and trace metals were analyzed. 

Multivariate analysis allows to increase the power of analysis and to inspect different relations between traits, especially for polygenic traits when one locus may have an opposite effect on different traits of interest. For instance, a certain locus may positively affect the concentration of one element and negatively affect the concentration of another. This approach is widely used in animal breeding and human genetics but not widely used in plant genomics [20,21,22,23,24]. 

In this study, we performed a multivariate genome-wide association study on the dataset of the common Russian wheat lines *Triticum aestivum*. Seven traits that are the concentration levels of Zn, Mg, Mn, Ca, Cu, Fe, and K in grain were studied. As multivariate analysis allows for the investigation of combinations of traits, it becomes useful in breeding because it enables researchers to avoid the cases when one concentration increases and another decreases.

## 2. Material and Methods

### 2.1. Wheat Lines

A panel of 157 common spring wheat genotypes was used in this study. The panel consisted of 105 bread wheat varieties, 48 introgression lines (ILs), and 3 wheat relatives (*Triticum dicoccum*, *Triticum timopheevii*, and *Triticum kiharae*). Introgression lines were developed on the base of hybridization of spring bread wheat varieties with wild relatives (*T. durum*, *T. dicoccum*, *T. dicoccoides*, and *T. timopheevii*) and synthetic hexaploid wheat *T. kiharae*. More information on the origin of accessions including a list of plant material is available in [25].

### 2.2. Genotyping and Quality Control

Genomic DNA was isolated from 5–7 day-old seedlings as described in [26]. DNA purification for SNP genotyping was performed using the “Bio-Silica kit for DNA purification from reaction mixtures” according to the manufacturer’s protocol. DNA was then quantified using Qubit dsDNA BR Assay kits (Thermo Fisher Scientific, Waltham, MA, USA) on a Qubit 4 Fluorometer (Thermo Fisher Scientific). SNP genotyping was performed using the Illumina Infinium 15 K Wheat platform by TraitGenetics-Section of SGS Institute Fresenius GmbH (Gatersleben, Germany, www.traitgenetics.com (accessed on 1 August 2023)). 

The dataset of 157 wheat lines, which included 13,007 SNP markers, was mapped on the wheat genome [27]. The genotypes were converted in the BED Plink format [28]. In total, 157 lines and 12794 SNPs were available for quality control and further analysis. We performed filtering of the lines and SNPs using the following filters: call rate of SNPs < 5% (--geno 0.05), minor allele frequency (MAF) < 1% (--maf 0.01), and call rate of lines < 5% (--mind 0.05). After filtering, 149 wheat lines and 11,405 SNPs remained. 

The chromosomes 1A, 1B, 1D…7A, 7B, and 7D were labeled as 1, 2, 3, …, 19, 20, 21, respectively. The “Unknown” chromosome was labeled as 22. The distribution of SNPs along chromosomes (Supplementary Figure S1) shows that SNPs are distributed unequally, and many SNPs were located on the “Unknown” chromosome.

PCA analysis was performed using plink (v.1.90b6.26, [28]), and PCA plot was generated in R (version 2022.07.0, build 548) using ggplot2 and ggrepel libraries. 

### 2.3. Phenotyping

Wheat genotypes were planted on the field of the Federal Research Center Institute of Cytology and Genetics of Siberian Branch of the Russian Academy of Sciences (Novosibirskaya Oblast, 54.9191° N, 82.9903° E) in 2018 and 2019. The experiment was conducted using randomized block design in two replicates on plots of 1 m width, 80 grains per row, and between-row spacing of 25 cm. Samples were sown in the second half of May and harvested in September in the phase of full grain ripeness. The soil of the experimental field consisted of leached chernozem; the fertile soil layer varied within 40–60 cm, and the humus content was 4.2%. The contents of nitrogen, phosphorus, and potassium were 0.34%, 0.30%, and 0.13%, respectively.

The growing season of 2018 was characterized by low temperatures in May (on average, 5 °C below normal) and high waterlogging in May–June. The weather conditions in 2019 were unstable due to uneven precipitation and temperature fluctuations in the second half of the growing season. Rainy weather was observed in May and July of 2019, and a slight drought was observed in June and August. 

Randomly selected 20 spikes were harvested and bulk-threshed manually, and 10 g of grain from each genotype was used for evaluation of seven macro- and microelements (Zn, Mg, Mn, Ca, Cu, Fe, and K). The element contents were measured by atomic absorption spectrometry with flame atomization on a ContrAA 800 D instrument (Analytik Jena, Germany). Statistical processing of the results was performed using the Statistica v.10.0 software package.

We used data on the concentration of seven different elements in grain—Zn, Mg, Mn, Ca, Cu, Fe, and K—measured consequently in 2018 and 2019 for each wheat line. There were two replicas for each line and year for a total of 157 × 2 × 2 phenotypic measurements. For each element measurement in each year, we removed values outside the range of 3 IQR. Visualization and descriptive statistics were performed in R. Heritability (h^2^) was calculated using REML analysis implemented in GCTA (v1.94) [29]. Proportion of the explained variance for each of the elements was calculated using ANOVA in R. In this analysis, we considered the effect of the year or line on the element concentration separately. 

### 2.4. GWAS Analysis 

As for each wheat line the phenotypic information about two replicates for each year was available, we represented each line as a “twin pair”, where genotypes were the same while phenotypes were different related to each of the samples. Thus, we performed GWASs using 149 × 2 lines for each year. This approach gives an opportunity to increase the sample size and consequently the power of the study. GWAS analysis for each year and concentration level of the element was performed using mixed model approach implemented in GCTA (v1.94) [29]) fastGWA (fast MLM-based Genome-Wide Association) tool with the parameters --maf 0.01 (MAF ≥ 1%), --geno 0.05 (SNP call rate ≥ 95%), and --mind 0.05 (sample call rate ≥ 95%). Also, we used parameter --allow-no-sex (as there was no information about sex for samples). 

We meta-analyzed the results for each concentration for two years to obtain a total sample size of 149 × 2 × 2 lines. Z-based sample-size-weighted meta-analysis was performed using the METAL (version 2011-03-25) [30]. 

As the next step, we combined different concentration levels into four multivariate traits and performed a multivariate GWAS. Multivariate traits clusters were formed based on the correlation matrix between traits (Supplementary Figure S2): (1) Ca and K; (2) Fe and Mn; (3) Mg and Zn; and (4) all elements. Beta and SE for beta used in the following multivariate analysis were calculated from METAL Z-scores according to the formula from [31]. Multivariate analysis was performed in R using the MultiABEL library. All genotyped SNPs were taken as a list of independent SNPs. 

The *p*-value threshold for meta-analysis and multivariate analysis was set at 4.01 × 10^−7^. This value was obtained by the formula (0.05/(11,334 × (7 + 4)), where 11,334 is the number of SNPs used in the analysis, 7 is the number of univariate GWASs, and 4 is number of multivariate GWASs. Lambda values for each GWAS analysis were calculated in R. Variance of the elements explained by each significant SNP was estimated using *p*-value and number of genotypes involved in meta-analysis for traits. We used function qchisq (*p*-value, df = 1, and lower.tail = False) in R to obtain a chi-square value with df = 1 and then divided it by the number of genotypes. The result was multiplied by 100 to receive variance of the elements explained by SNP in percent. 

For QQ plots, R library qqman (v.0.1.8) was used, and for Manhattan plots and phenotypic correlation matrix (with a correction for kinship, correlation method ward.D), we used R libraries RcolorBrewer (v.1.1.3), corrplot (v.0.92), qqman (v.0.1.8), and dplyr (v.1.1.1). 

### 2.5. Functional Annotation

The gene nearest to a significant loci was determined with the EnsemblPlants genome browser [32] using track Sequence Genes. Detailed gene description was obtained from the UniProt database [33], and the level of certainty for protein annotation for all genes was labeled as “Predicted”.

### 2.6. Verification of Known Loci

We compared results from meta-analysis for each element and from clusters of multivariate analysis with already published results from [11,13]. First, we took all significant loci from the cited papers. Second, we verified whether these loci were in our analyzed dataset. Third, we considered whether the *p*-values for these loci obtained from the meta- and multivariate analyses were significant; i.e., *p*-value was below 0.05/35, where 35 is the number of significant loci from the papers.

## 3. Results

### 3.1. Concentration of Elements in Grain

We checked the distributions of the element concentration levels for each year (Supplementary Figures S3–S9). Some of the phenotypes had a skewed normal distribution. Up to 30 outliers were observed for each trait taking together two replicates for each year. These observations reveal the degree of diversity in the studied lines (Supplementary Table S1).

Heritability estimates (h^2^) were moderate almost for all traits (except Ca in 2018 and 2019, Mg in 2019, and Zn in 2019) with a good resemblance of estimates between years (Table 1). The proportion of the explained variance of element concentrations was high when we considered the differences between lines (Supplementary Table S2)—from 0.345 to 0.757. But when we considered the effect of the year, the explained variance was much lower—from 0.009 to 0.303. 

### 3.2. Genetic Structure of the Studied Populations/Lines

**Figure 1:**
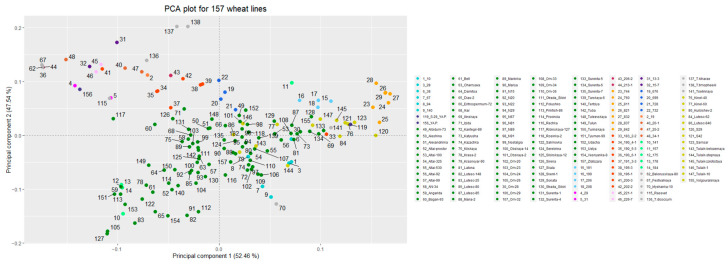
PCA plot describing population structure of the studied wheat lines. Colors correspond to different geographical areas of the origin, and numbers on the PCA correspond to the line names provided in the legend.

The PCA plot of the 157 genotypes is presented on Figure 1. Fifteen different areas are illustrated by different colors, and every wheat line on the PCA is labeled by a number (described in the legend). As it can be seen, samples from the same geographical areas are located next to each other with a few exceptions. In general, there are two main clusters on the PCA: the first forming an oval in the middle of the plot and the second, more dispersed, in the upper left corner. There are samples genetically close to each other, for instance, lines 195-3 and 196-1 (numbers 38 and 39 on the PCA) or a cluster of lines 190 6-1, 213-1, Belorusskaya-80, and Festivalnaya (numbers 36, 44, 62, and 67, respectively). The first 10 components of the PCA compose a usual slope as it was observed in many different studies (Supplementary Figure S10). 

### 3.3. GWAS of the Concentrations of Seven Elements

We performed a GWAS for each element and each year separately with the consequent meta-analysis of the GWAS for two years. In total, seven univariate GWAS results were obtained. Genomic control lambdas varied from 0.98 to 1.12 (Supplementary Table S3), with the GWAS for Mn having the biggest lambda. QQ plots showed deviations from the expected distribution for all the elements, except for Ca. Probably, this might be a result of the meta-analysis where an increased sample size led to an increased power of the analysis (Supplementary Figures S11–S17). The combined Manhattan plot for all seven concentration levels is presented in Supplementary Figure S18. One SNP (BS00022069_51) was significantly associated (*p*-value < 4.01 × 10^−7^) with the level of K. 

**Figure 2:**
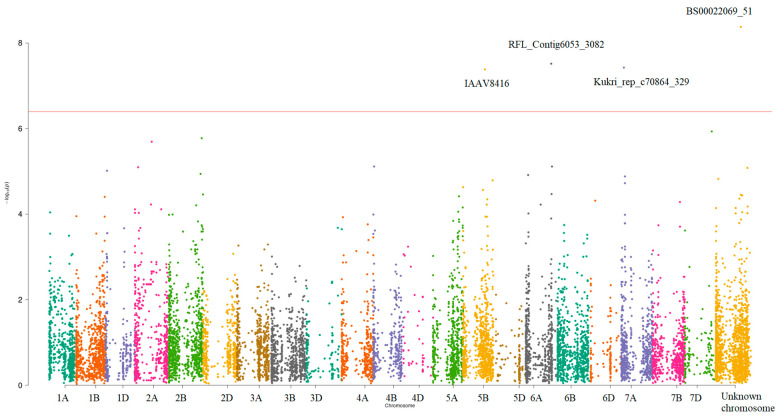
Manhattan plot for GWAS meta-analysis of the wheat line datasets. Four significant SNPs (*p*-value < 4.01 × 10^−7^) are located on the 5B, 6A, 7A, and Unknown chromosomes (for more details, see Table 2).

As the next step, we performed the analysis of four multivariate traits. The joint results for the univariate and multivariate GWASs are presented in Table 2. The joint Manhattan plot for multivariate traits is presented in Figure 2. Genomic control lambdas varied from 0.97 to 1.10 (Supplementary Table S4). QQ plots showed deviations from the expected distribution for all multivariate traits (Supplementary Figures S19–S22). 

Four SNPs were significantly associated with at least one multivariate trait, with one of the SNPs (BS00022069_51) significantly associated with K levels. For more details, see Table 2.

### 3.4. Functional Annotation of Discovered Loci

We used the EnsemblPlants genome browser [32] to investigate the nearest to the discovered top SNPs genes. We determined the nearest gene for three loci. The top SNPs of these loci were located within coding exons of these genes (Table 2).

### 3.5. Replication of Known Loci

We replicated one known published locus associated with the Zn concentration (IAAV1375, *p*-value in meta-analysis 0.001065, in multivariate analysis in cluster Mg and Zn—0.0029 and in cluster with all elements—0.0020) discovered in [11] (Supplementary Table S5). In the mentioned as well in the current study, the IAAV1375 genotype CC was associated with an increased Zn concentration in grain.

## 4. Discussion

In this study, we performed a multivariate genome-wide association study on Russian lines. We replicated one already known locus (IAAV1375) and discovered four novel loci that reached the stringent Bonferroni-corrected genome-wide significance. One of these loci was detected both in the meta- and multivariate analyses, while three others were detected in the multivariate analysis only. 

The know replicated locus IAAV1375 is located on the chromosome 5A, position 502220915 inside the intron of TraesCS5A02G291300 protein-coding gene. According to [11], this gene is a probable UDP-arabinose 4-epimerase 1. We searched for orthologs of this gene in PantherDB [34] and found numerous orthologs belonging to the oxidoreductase PANTHER protein class in different plants. Furthermore, by utilizing the PANTHER Family and PANTHER Subfamily classifications, we obtained a more specific description: PINORESINOL REDUCTASE-RELATED and NMRA DOMAIN-CONTAINING PROTEIN. Unfortunately, no additional information was available for any of the plants with orthologs of this gene.

Interestingly, according to the novel wheat genome assembly (RefSeq v1.0), locus IAAV1375 is located on the same chromosome as locus BS00022069_51, which was found in our discovery analysis. These loci are relatively distant from each other (79.3 Mb). However, they exhibit a small but significant nonzero linkage disequilibrium (R^2^ = 0.09, D’ = 0.51). We can speculate that this chromosomal region (chromosome 5A, positions 502 Mb–582 Mb) may contain a cluster of genes related to the regulation of element concentrations.

For all revealed loci, we determined the nearest genes, and, surprisingly, three of them were located in protein-coding genes. Moreover, the coordinates of top SNPs (see Table 2) were located within coding exons. A significant SNP from the RFL_Contig6053_3082 locus was located in the gene TraesCS6A02G375400. According to the UniProt, this gene belongs to ENTH domain-containing proteins playing a role in endocytosis and cytoskeletal machinery. Another SNP from the Kukri_rep_c70864_329 locus was located within the TraesCS7A02G094800 protein-coding gene. This gene belongs to the CRAL_TRIO_N domain-containing proteins that has a spectrum of various functions. The third SNP from the IAAV8416 locus was located within the protein-coding gene TraesCS5B02G325400, but there is no information about its function. The fourth SNP from the BS00022069_51 locus was located within the protein-coding gene TraesCS5A02G384200, but there was also no information about its function.

We used Z-score-based meta-analysis in our pipeline. Consequently, the resulting effect had no scale, and we could interstate it only in terms of the effect’s direction. For five discovered loci (BS00022069_51, RFL_Contig6053_3082, Kukri_rep_c70864_329, IAAV8416, and IAAV1375), we specified positive alleles associated with concentrations of elements. For the BS00022069_51—allele, C was positively associated with the K concentration; for the RFL_Contig6053_3082—allele, T was positively associated with concentrations of Fe and Mn; for the Kukri_rep_c70864_329—allele, T was positively associated with the concentration of Fe and allele C was positively associated with the concentration of Ca; for the IAAV8416 allele, T was positively associated with the concentration of Mn; and for the IAAV1375 allele, C was positively associated with the concentration of Zn. 

Multivariate analysis indeed increases the power of analysis. This approach is widely used in animal and human genetics but not in plants [20,21,22,23,24]. The multivariate approach helps not only to increase the power of analysis but also to control the directionality of the effect for several traits to avoid the opposite selection.

To conclude, we performed a multivariate genome-wide association study of seven concentrations of elements and discovered four new loci and replicated one known. 

## Figures and Tables

**Table 1:** Heritability (h^2^) and standard error (se, in parentheses) for each of the traits in studied wheat lines.

Element	2018	2019
Ca	0.66 (0.052)	0.51 (0.059)
Cu	0.80 (0.034)	0.80 (0.032)
Fe	0.87 (0.024)	0.75 (0.040)
K	0.72 (0.048)	0.838 (0.030)
Mg	0.74 (0.043)	0.55 (0.058)
Mn	0.83 (0.029)	0.79 (0.033)
Zn	0.83 (0.028)	0.67 (0.047)

**Table 2:** GWAS results for the meta-analysis and multivariate analysis (*p*-value < 4.01 × 10^−7^).

Marker Name	Chromosome	Positive/Negative Alleles	Position	The Most Significant *p*-Value	Variance Explained by SNP, %	Significant Traits	The Nearest Gene
BS00022069_51	Unknown *	A/C	582104154	5.40 × 10^−9^	5.75	Ca and K, K	TraesCS5A02G384200 **
RFL_Contig6053_3082	6A	C/T	597790267	3.05 × 10^−8^	5.22	Fe and Mn	TraesCS6A02G375400
Kukri_rep_c70864_329	7A	C/T **	57873213	3.76 × 10^−8^	5.18	All traits	TraesCS7A02G094800
IAAV8416	5B	T/C ***	509487915	4.13 × 10^−8^	5.22	Fe and Mn	TraesCS5B02G325400

* According to novel assembly RefSeq v1.0, this locus is located on 5A chromosome. ** For Ca, positive allele is T and negative is C. *** For Mn, positive allele is C and negative is T.

## Data Availability

Data available on request.

## Supplementary Materials

The following supporting information can be downloaded at: https://www.mdpi.com/article/10.3390/plants12173019/s1, Figure S1: Distribution of 11 405 SNPs along the chromosomes of 149 wheat lines, Figure S2: Correlated element clusters determined by multivariate analysis, Figure S3: Distribution of Zn concentration in wheat grains in 2018 and 2019, Figure S3: Distribution of Zn concentration in wheat grains in 2018 and 2019, Figure S4: Distribution of Mg concentration in wheat grains in 2018 and 2019, Figure S5: Distribution of Mn concentration in wheat grains in 2018 and 2019, Figure S6: Distribution of Ca concentration wheat grains in 2018 and 2019, Figure S7: Distribution of Cu concentration wheat grains in 2018 and 2019, Figure S8: Distribution of Fe concentration in wheat grains in 2018 and 2019, Figure S9: Distribution of K concentration in wheat grains in 2018 and 2019, Figure S10: Percentage of genetic variation explained by the first 10 principal components, Figure S11: Q-Q plot in meta-analysis for Fe, Figure S12: Q-Q plot in meta-analysis for K, Figure S13: Q-Q plot in meta-analysis for Mg, Figure S14: Q-Q plot in meta-analysis for Mg, Figure S15: Q-Q plot in meta-analysis for Zn, Figure S16: Q-Q plot in meta-analysis for Cu, Figure S17: Q-Q plot in meta-analysis for Ca, Figure S18: Manhattan plot for GWAS meta-analysis of wheat lines dataset, Figure S19: Q-Q plot for a Mg and Zn cluster determined in multivariate analysis, Figure S20: Q-Q plot for a Fe and Mn cluster determined in multivariate analysis, Figure S21: Q-Q plot for a Ca and K cluster determined in multivariate analysis, Figure S22: Q-Q plot for all the elements considered jointly in multivariate analysis. Table S1: Dispersion values of element concentration in studied samples, Table S2: Proportion of the explained variance of elements concentrations considering effect of year and line separately, Table S3: Lambda values from GWAS meta-analysis, Table S4: Lambda values from multivariate GWAS, Table S5: Replication of loci from [11,13]. In green is locus with significant *p*-value.
