# Supplementary material for: Multivariate Genome-Wide Association Study of Concentrations of Seven Elements in Seeds Reveals Four New Loci in Russian Wheat Lines
Source: Plants (Basel). 2023 Aug 22;12(17):3019. doi: 10.3390/plants12173019 (PMC10489822; doi:10.3390/plants12173019)
Supplement: Supplementary file 1 [file plants-12-03019-s001.zip › Supplementary Figures Wheat GWAS.pdf]

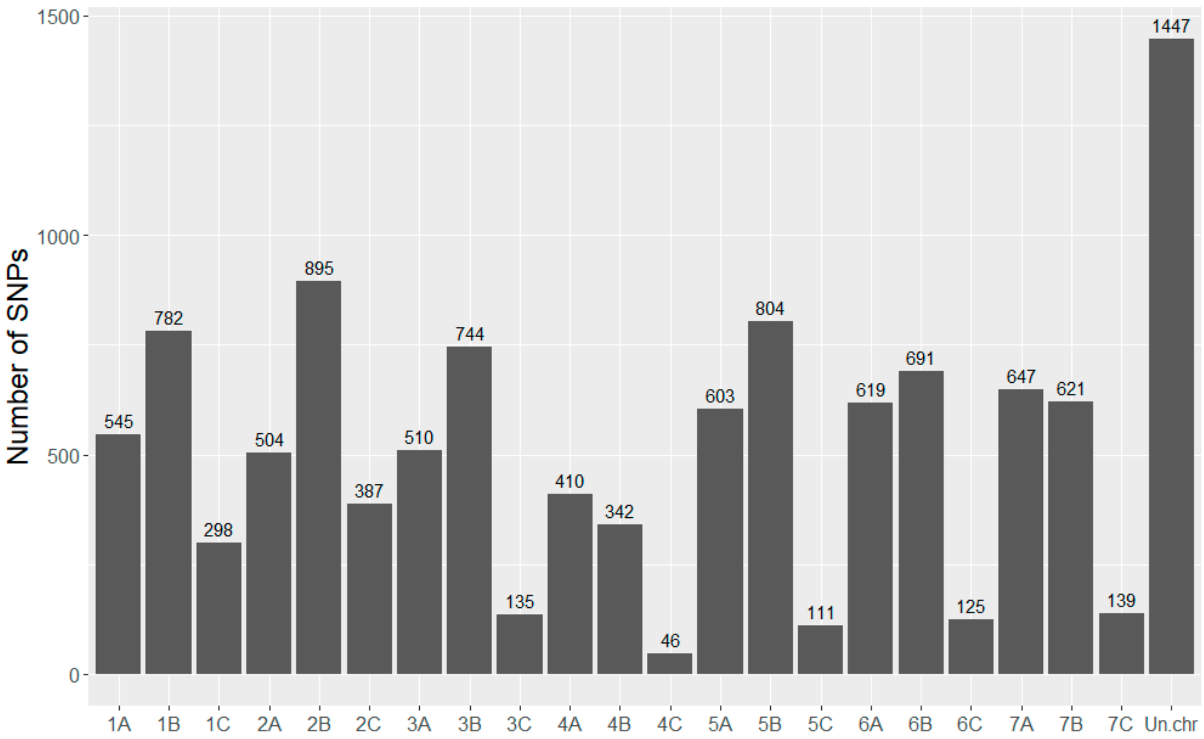

Supplementary Figure S1. Distribution of 11 405 SNPs along the chromosomes of 149 wheat lines.

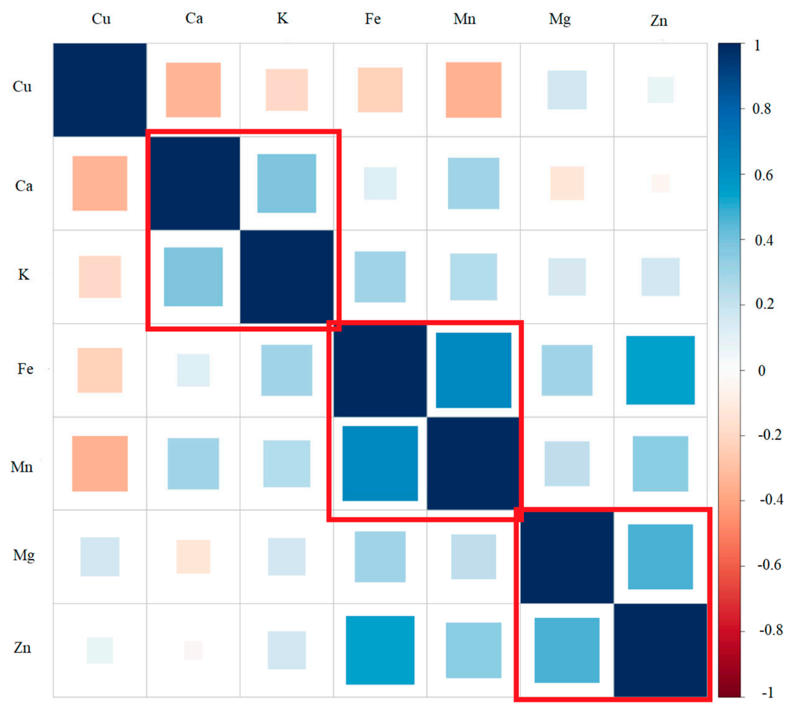

Supplementary Figure S2. Correlated element clusters determined by multivariate analysis.

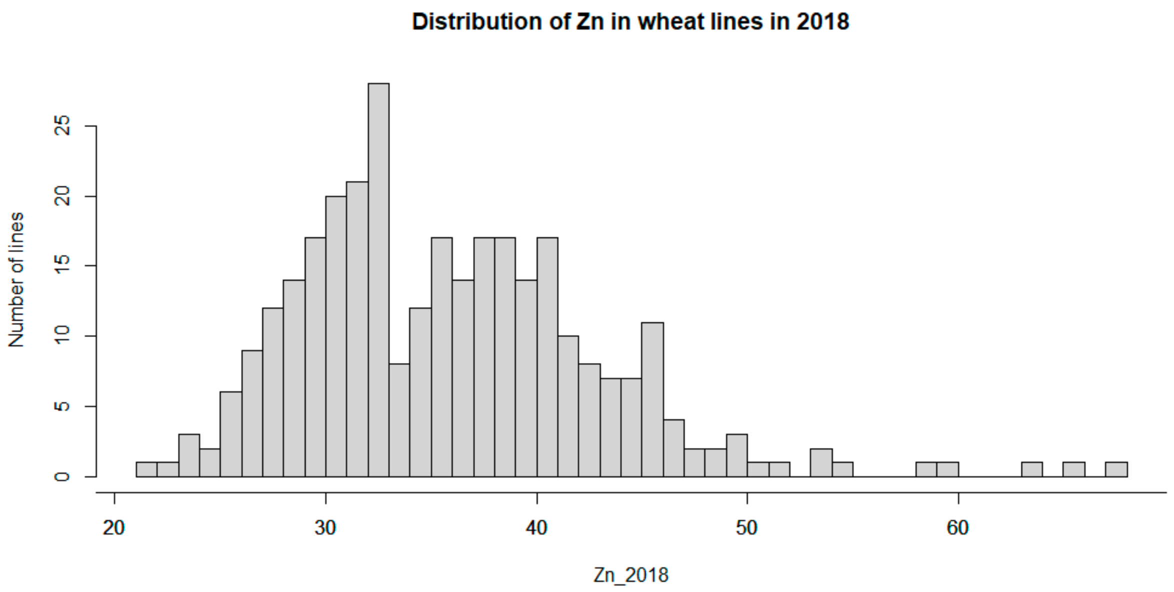

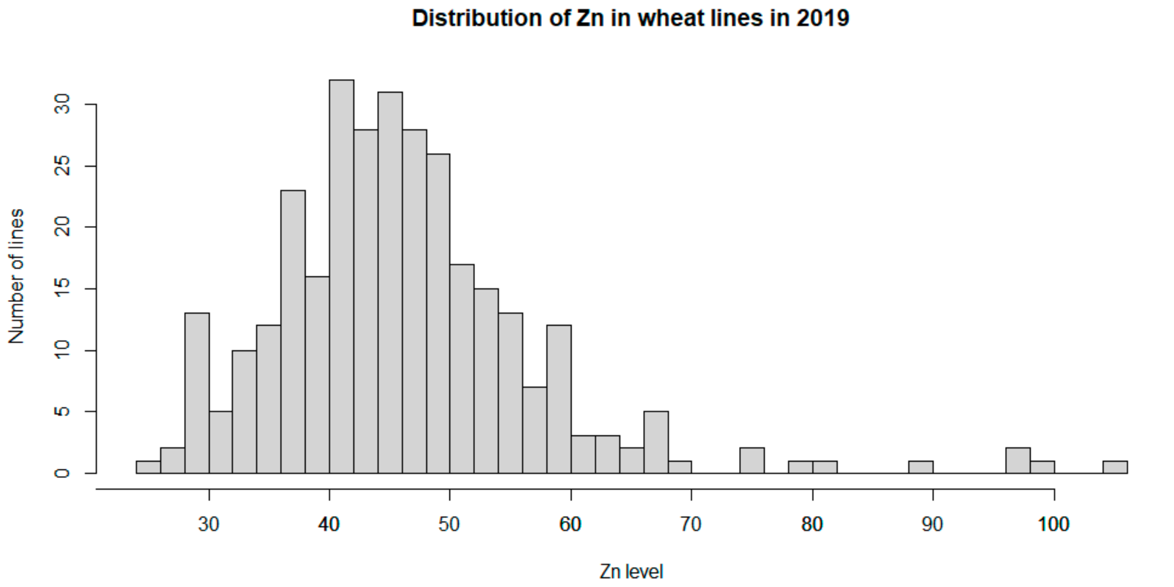

Supplementary Figure S3. Distribution of Zn concentration in wheat grains in 2018 and 2019.

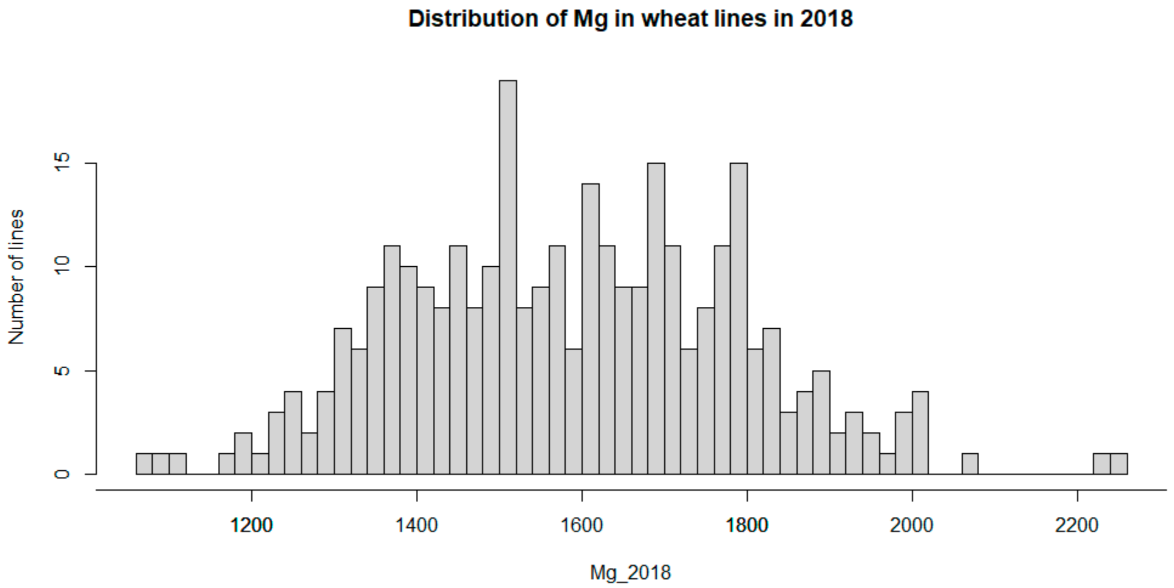

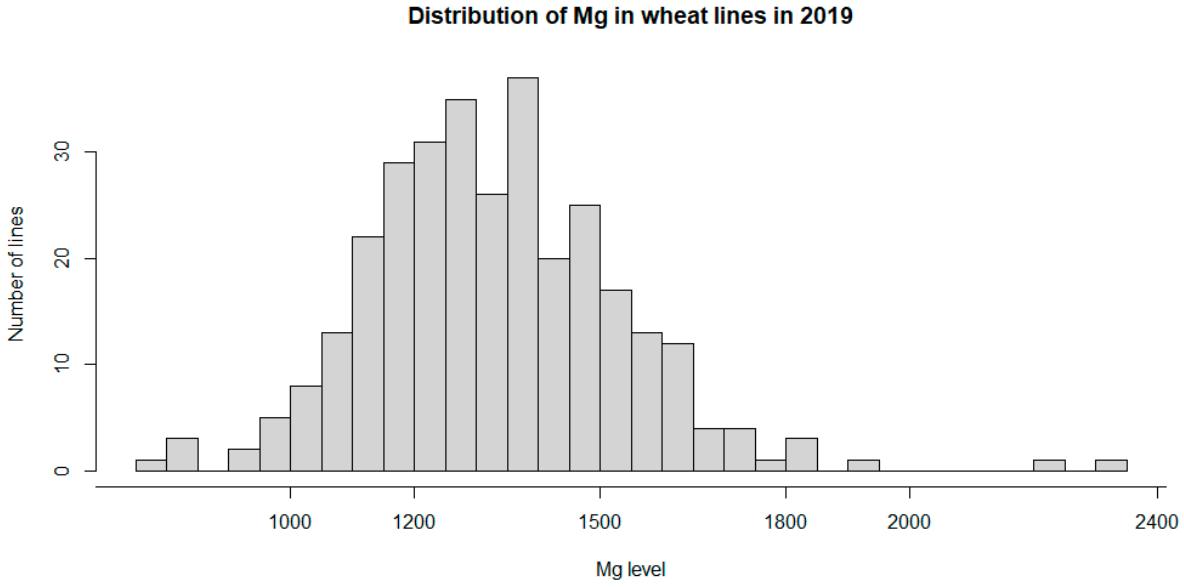

Supplementary Figure S4. Distribution of Mg concentration in wheat grains in 2018 and 2019.

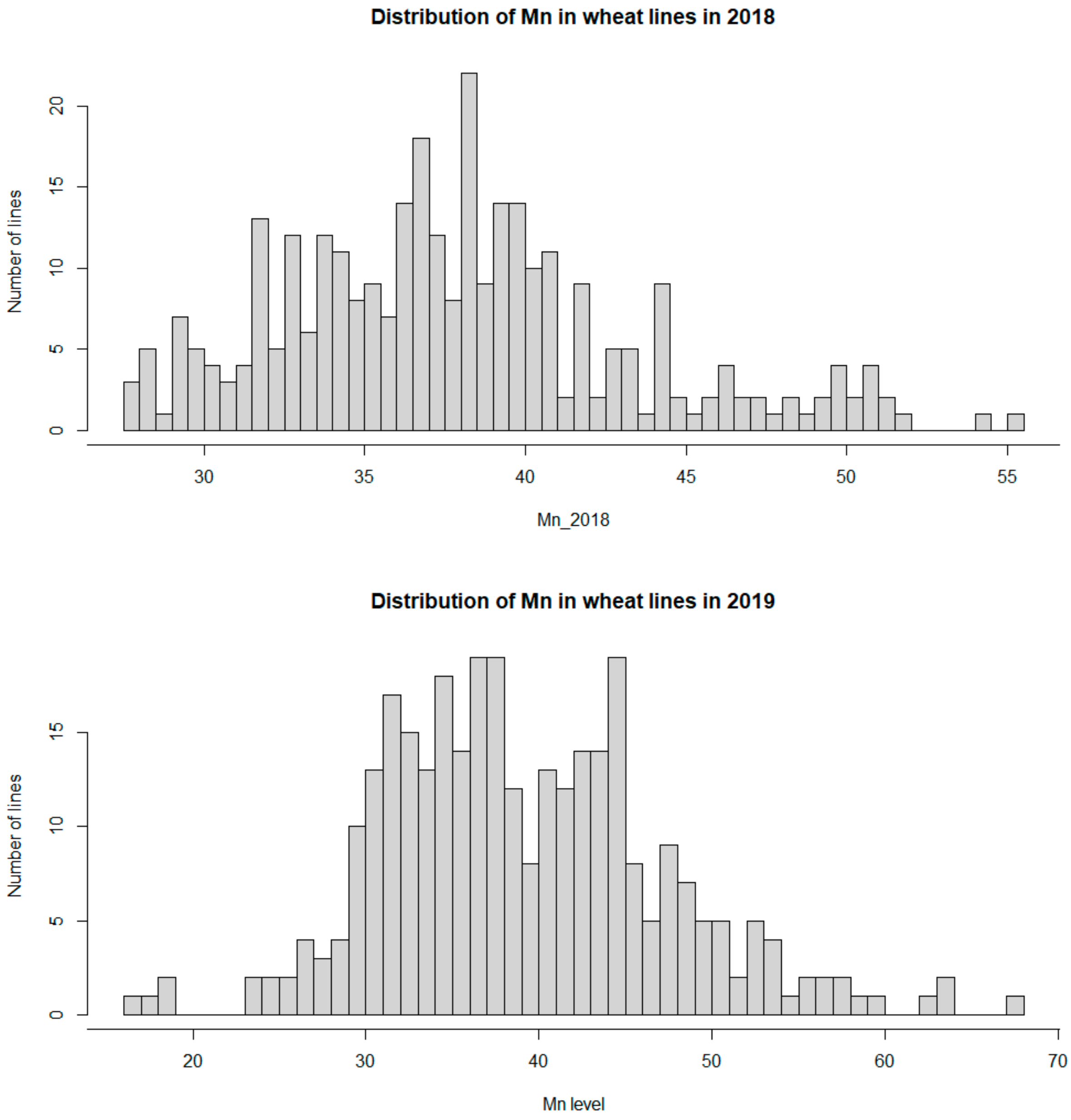

Supplementary Figure S5. Distribution of Mn concentration in wheat grains in 2018 and 2019.

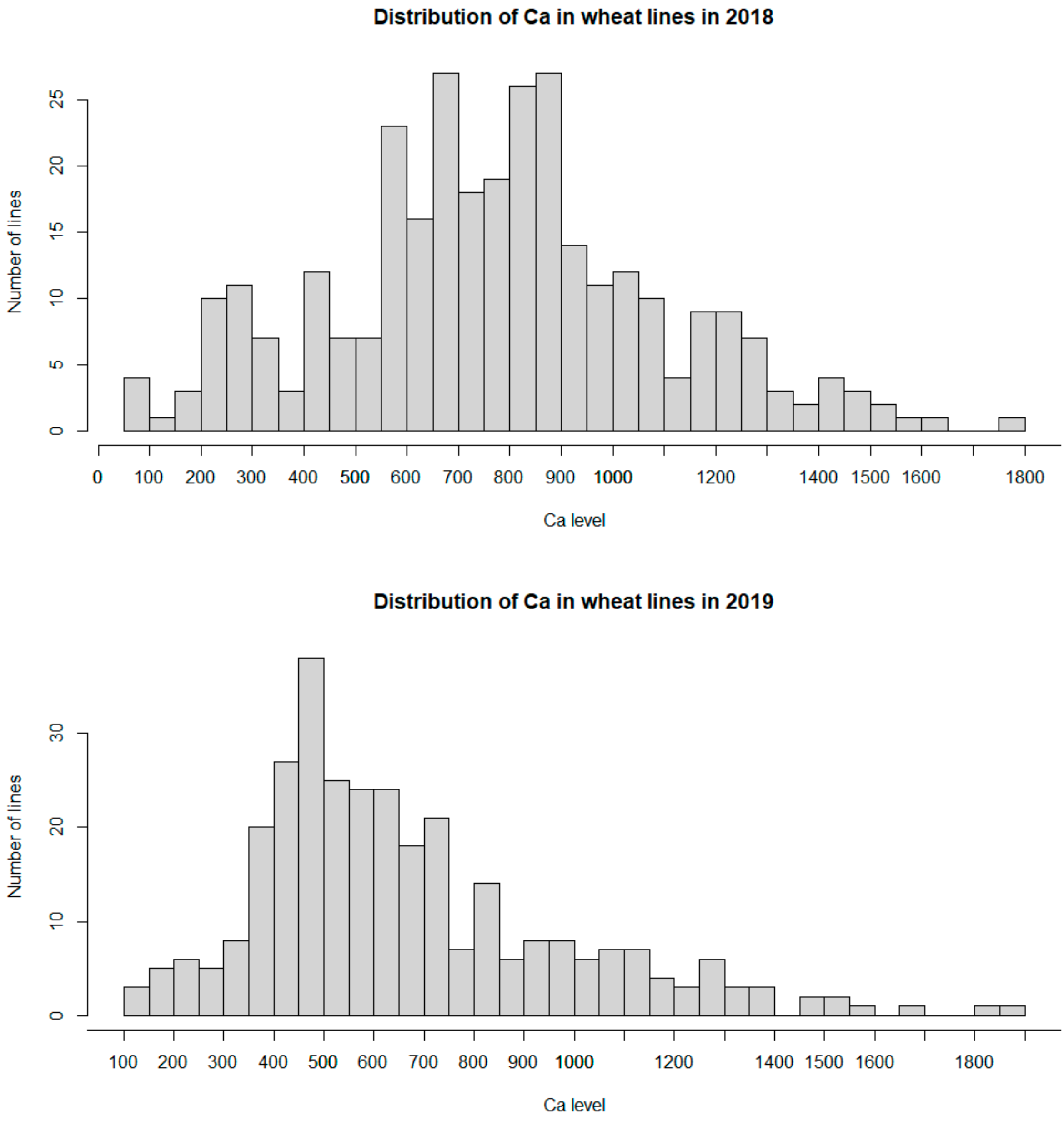

Supplementary Figure S6. Distribution of Ca concentration wheat grains in 2018 and 2019.

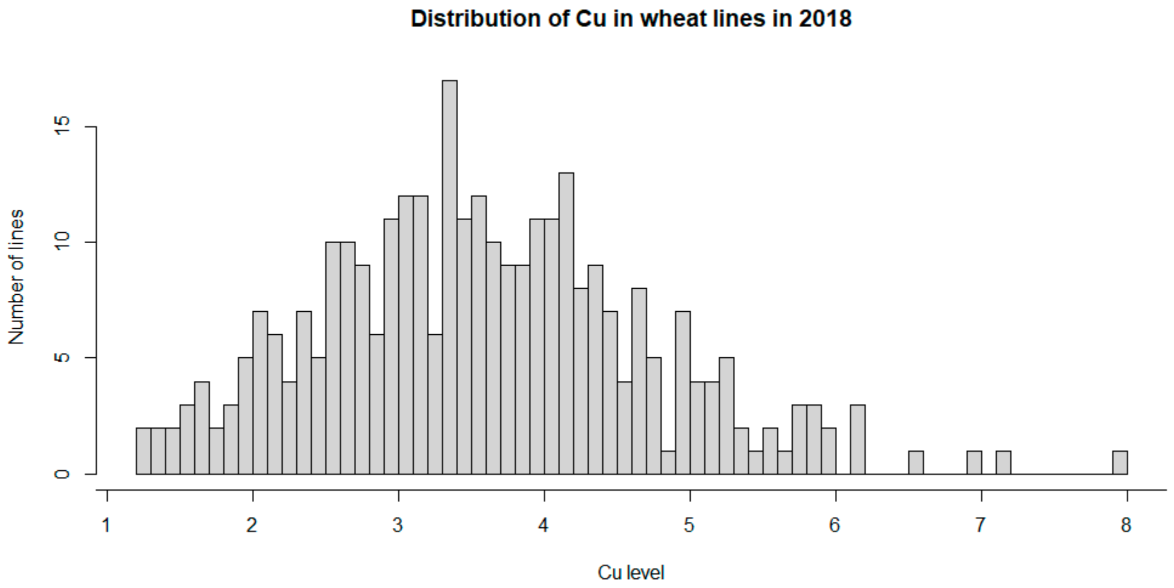

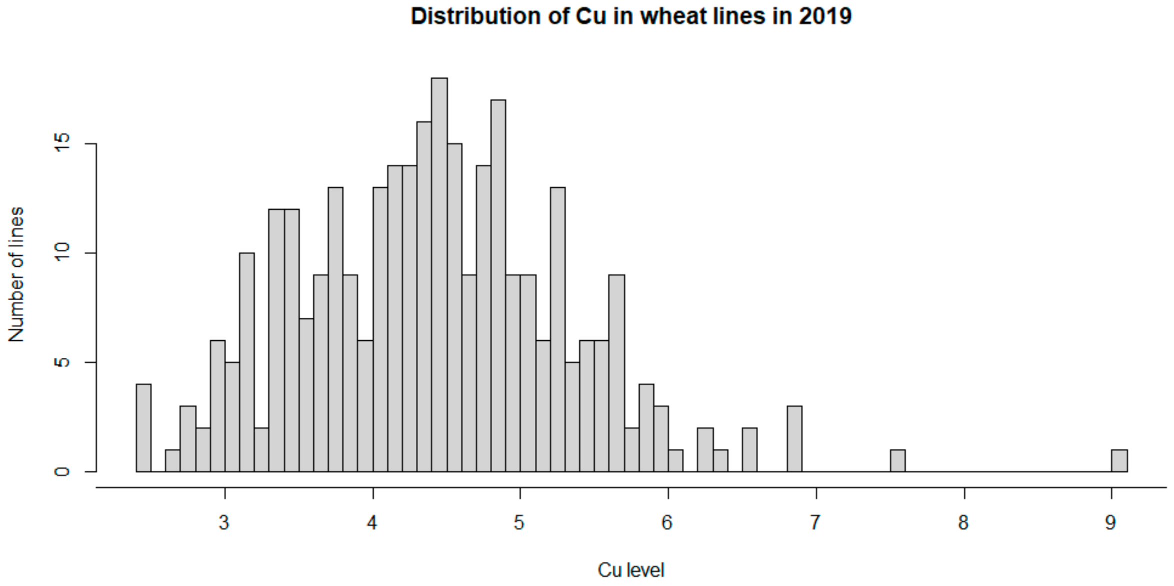

Supplementary Figure S7. Distribution of Cu concentration wheat grains in 2018 and 2019.

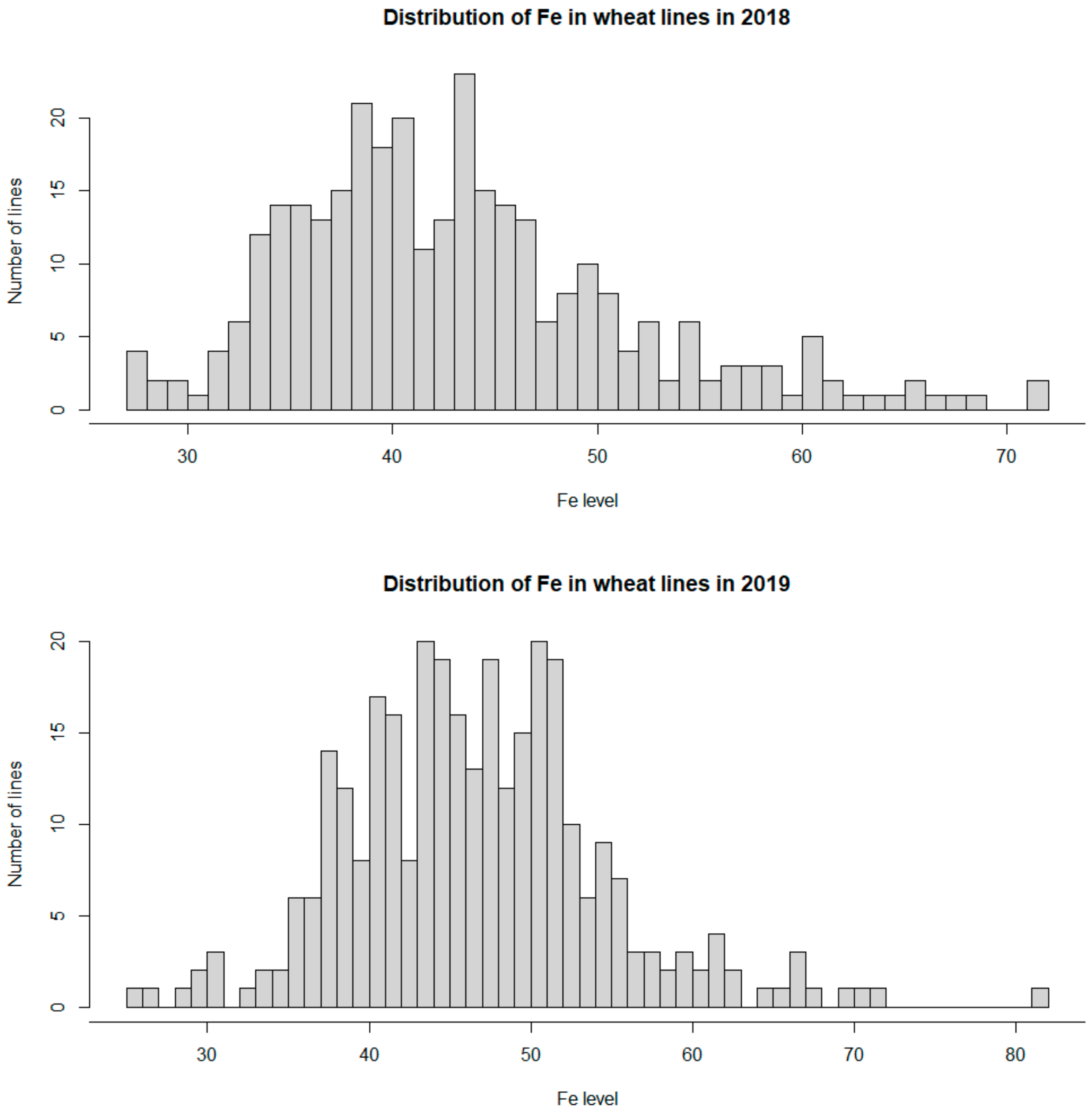

Supplementary Figure S8. Distribution of Fe concentration in wheat grains in 2018 and 2019.

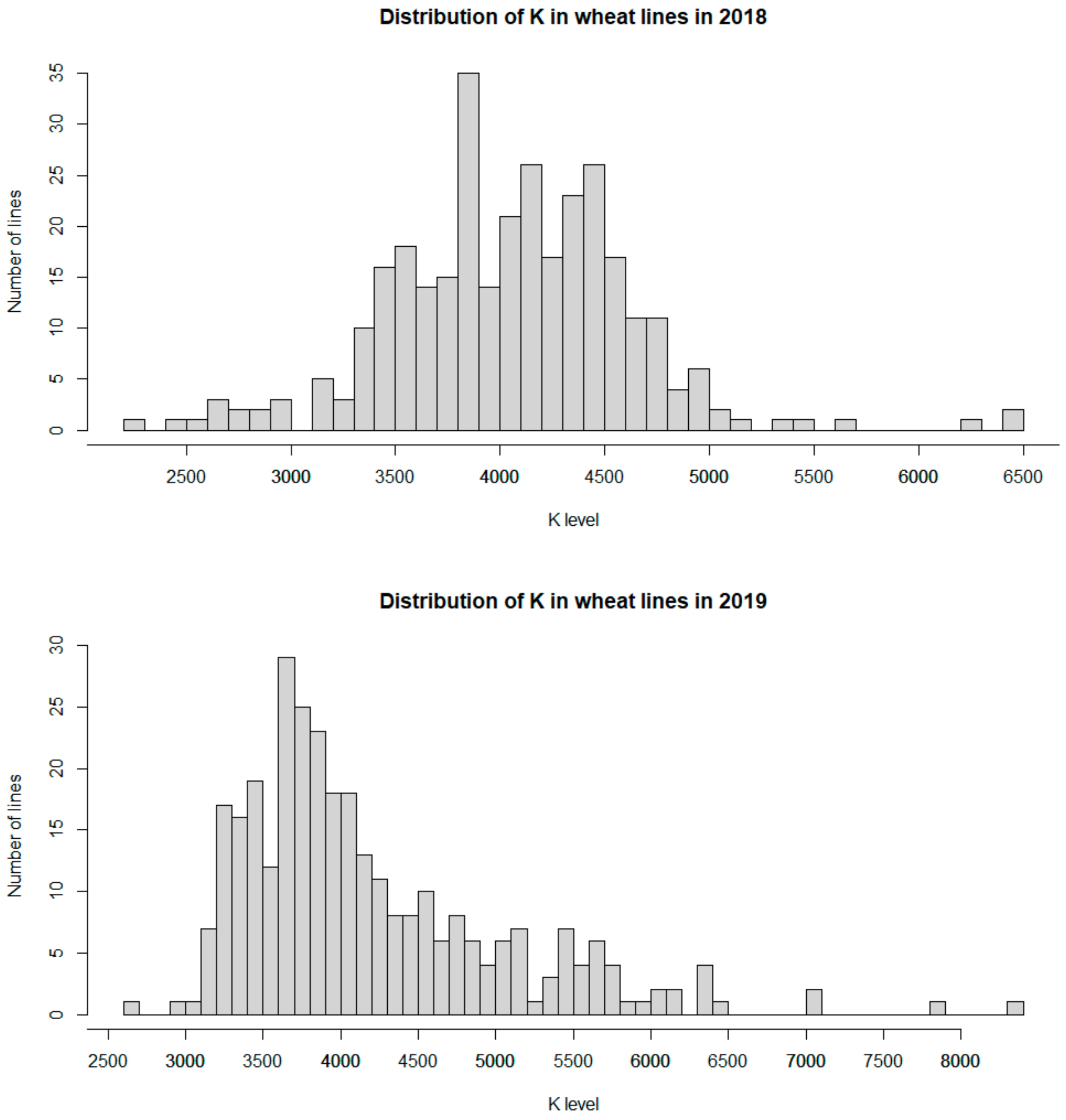

Supplementary Figure S9. Distribution of K concentration in wheat grains in 2018 and 2019.

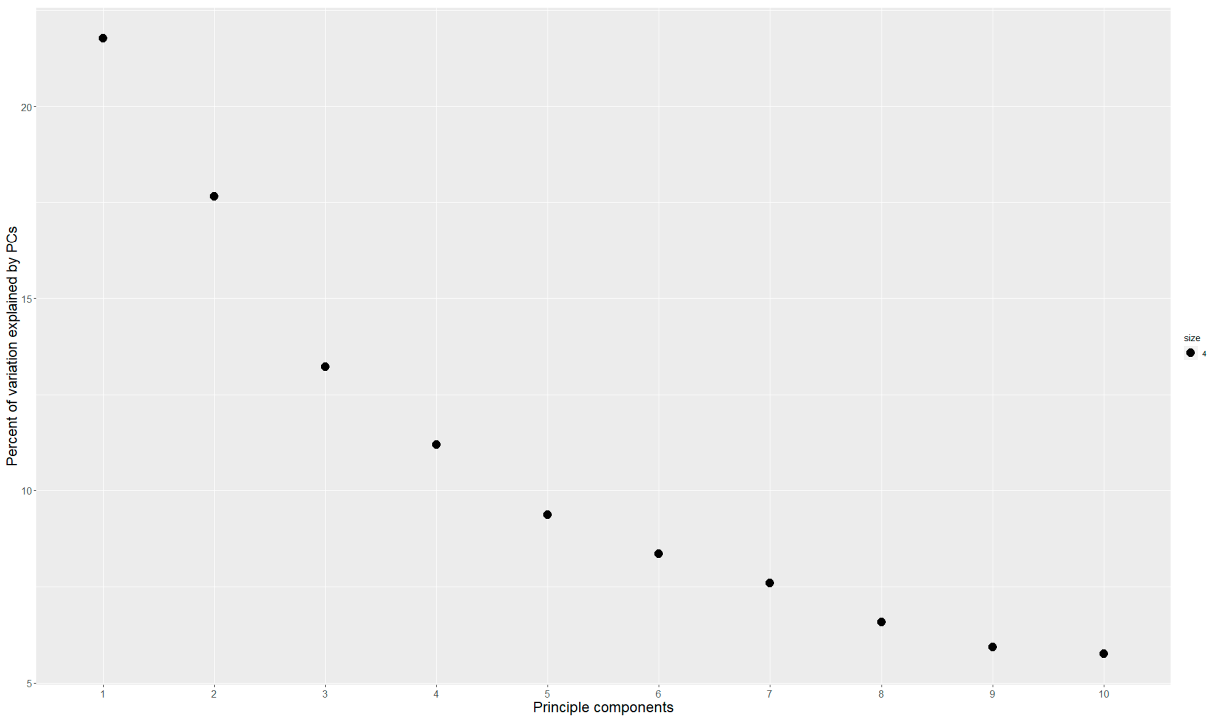

Supplementary Figure S10. Percentage of genetic variation explained by the first 10 principal components.

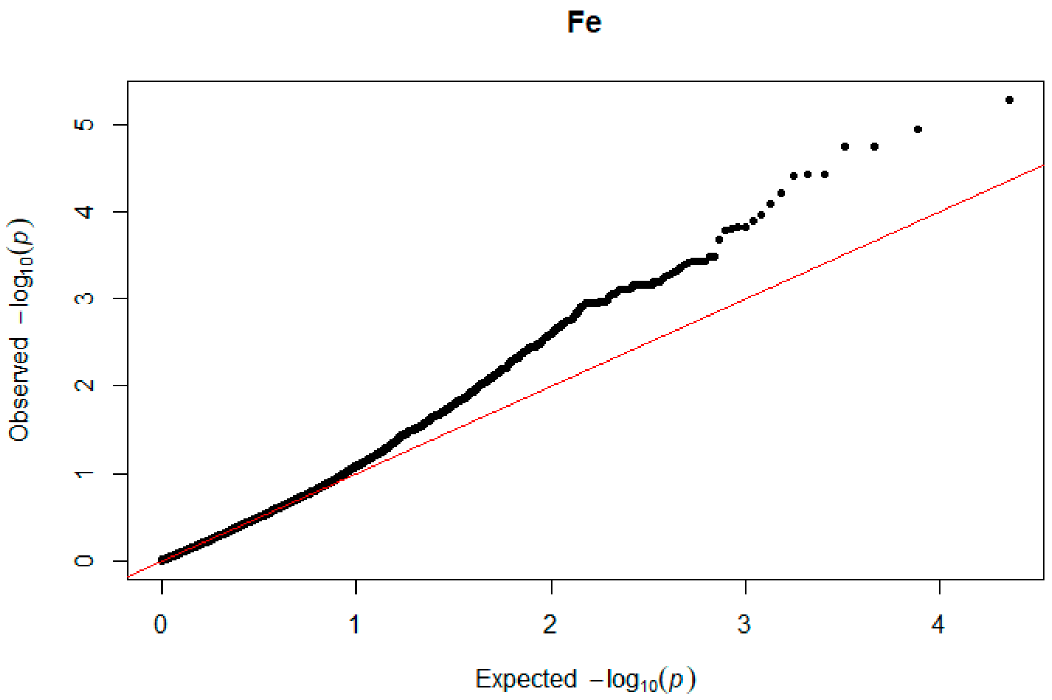

Supplementary Figure S11. Q-Q plot in meta-analysis for Fe.

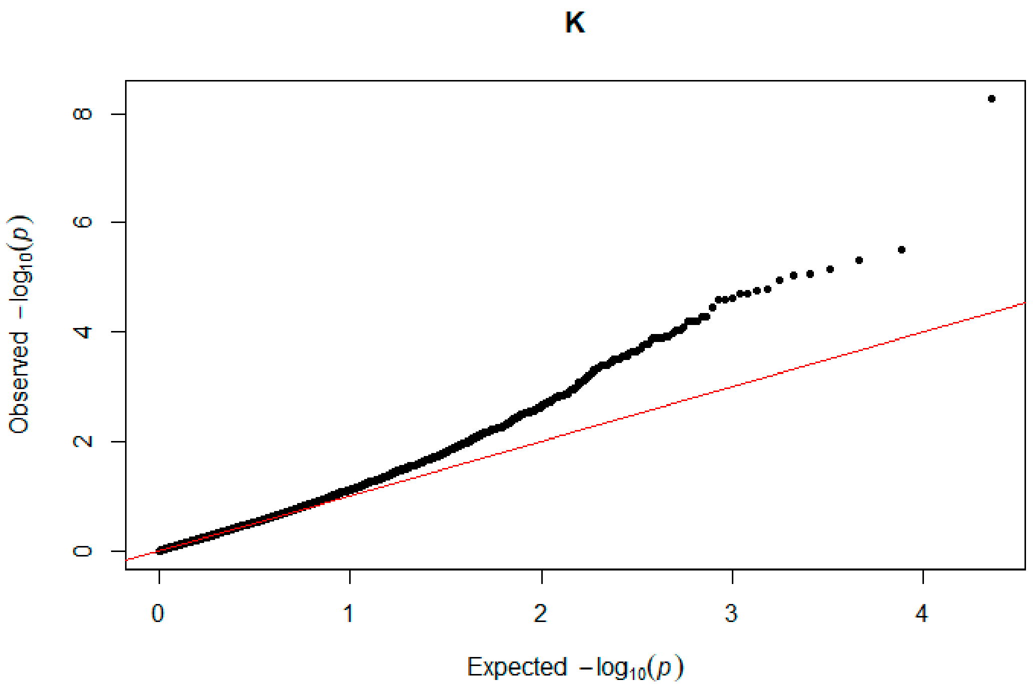

Supplementary Figure S12. Q-Q plot in meta-analysis for K.

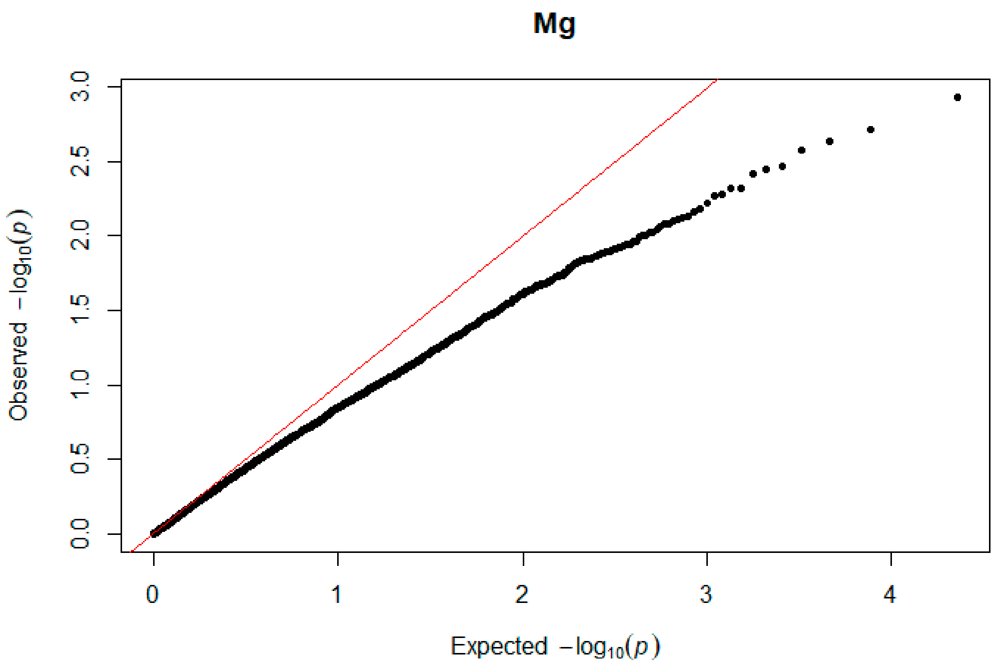

Supplementary Figure S13. Q-Q plot in meta-analysis for Mg.

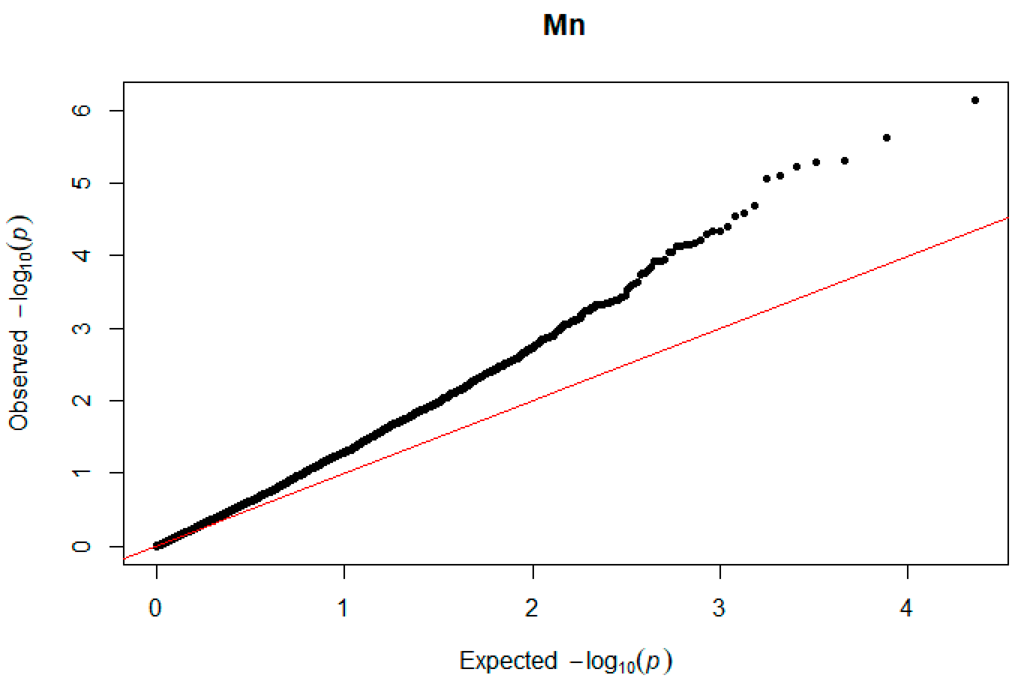

Supplementary Figure S14. Q-Q plot in meta-analysis for Mg.

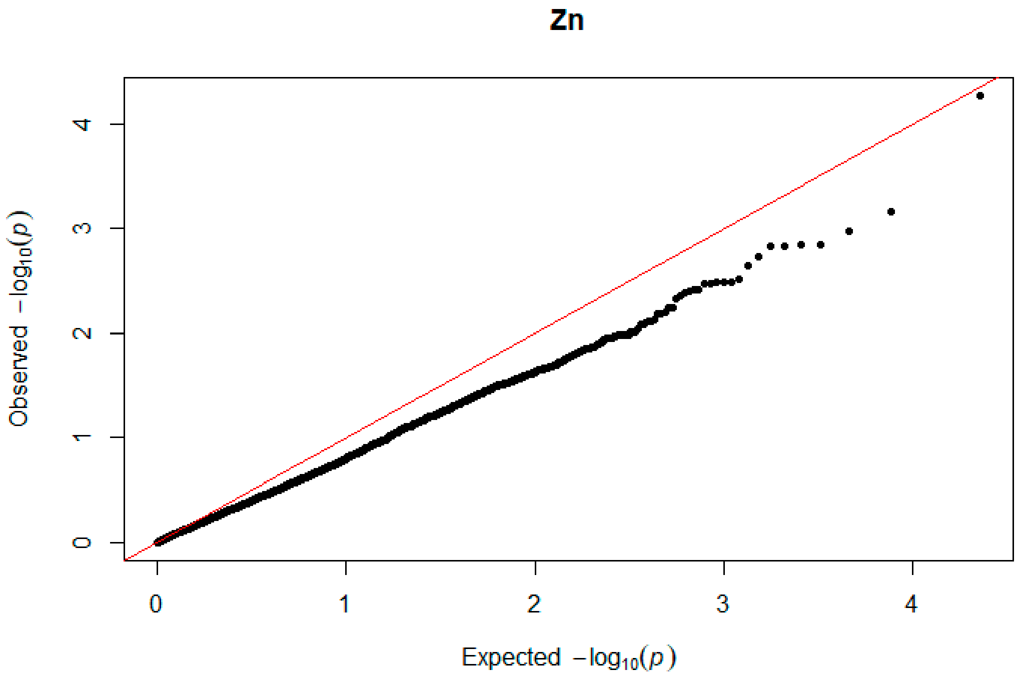

Supplementary Figure S15. Q-Q plot in meta-analysis for Zn.

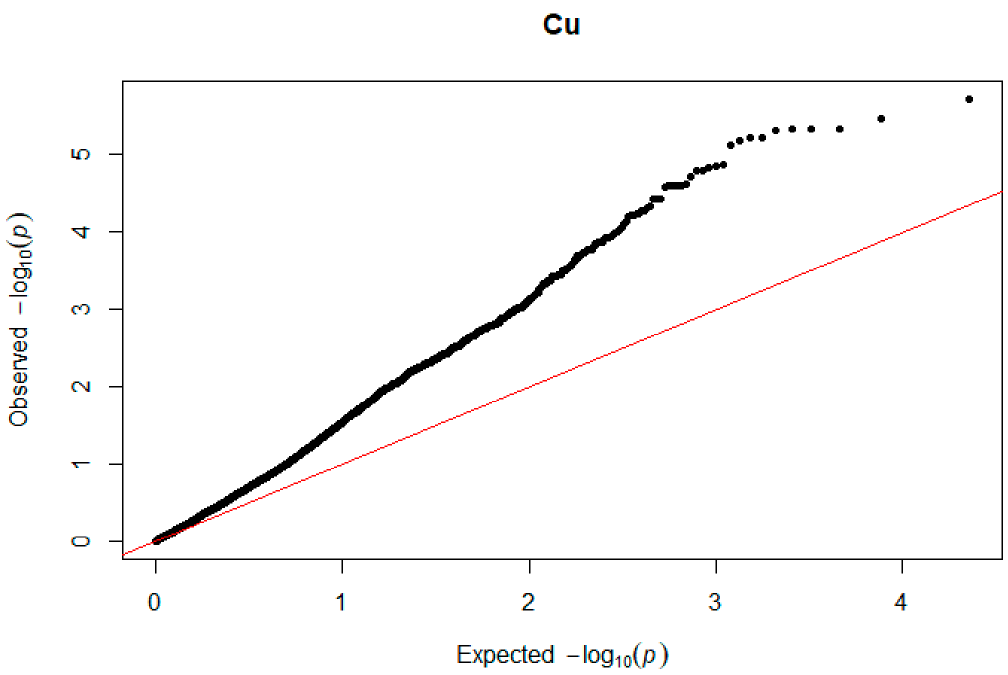

Supplementary Figure S16. Q-Q plot in meta-analysis for Cu.

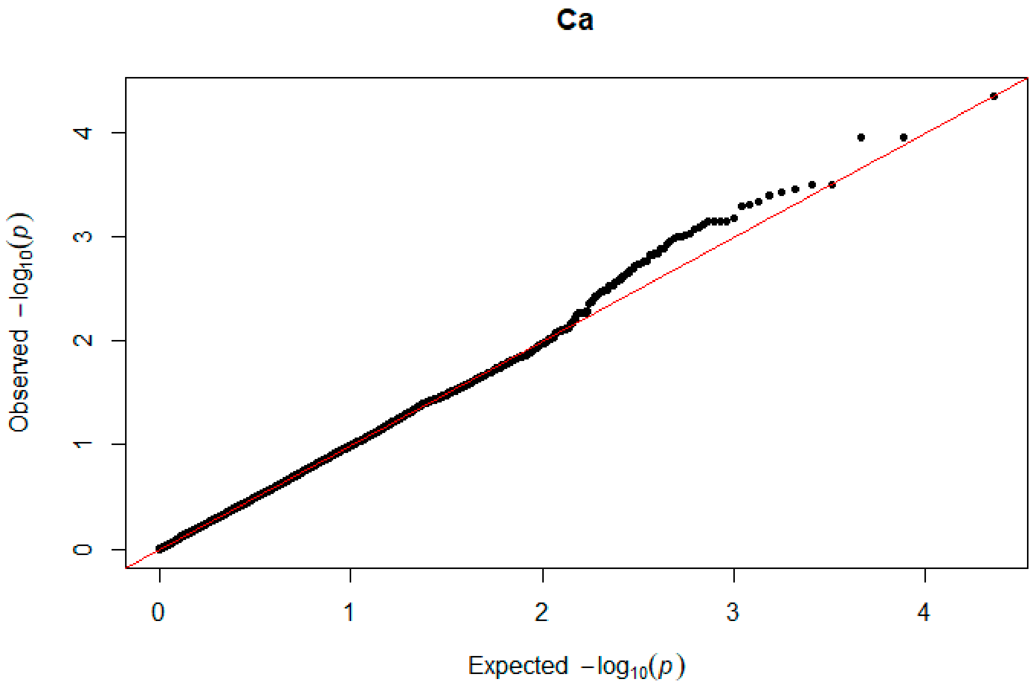

Supplementary Figure S17. Q-Q plot in meta-analysis for Ca.

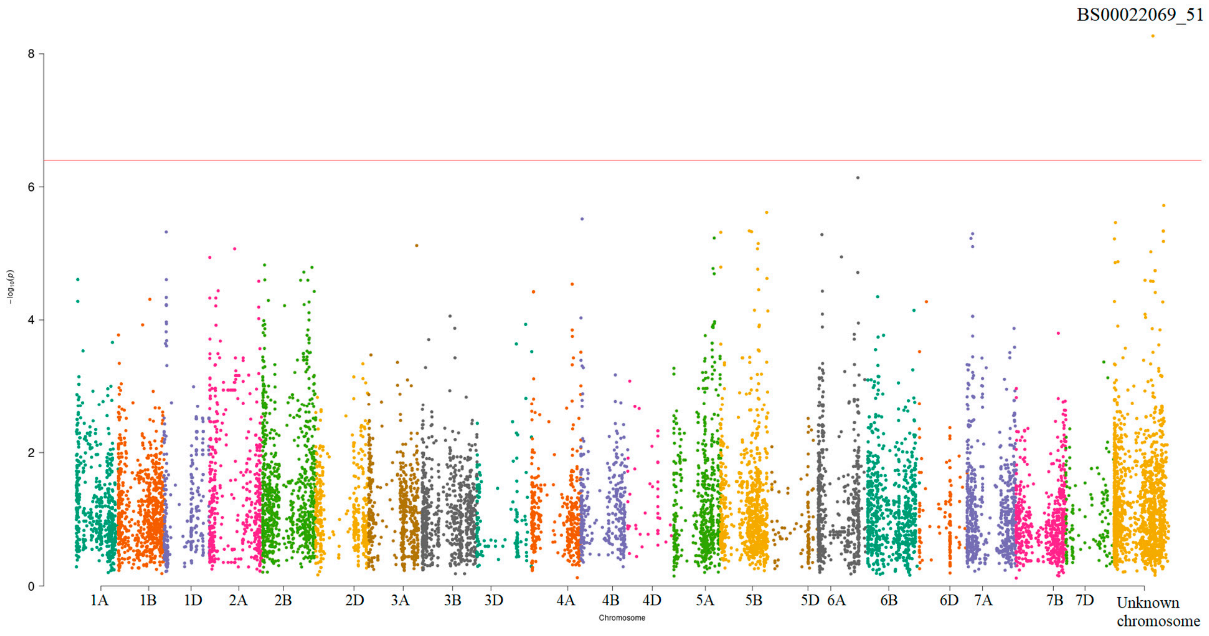

Supplementary Figure S18. Manhattan plot for GWAS meta-analysis of wheat lines dataset. The only one significant SNP (p-value < 4.01e-7) is located on the Unknown chromosome and shows association with K level in the grain.

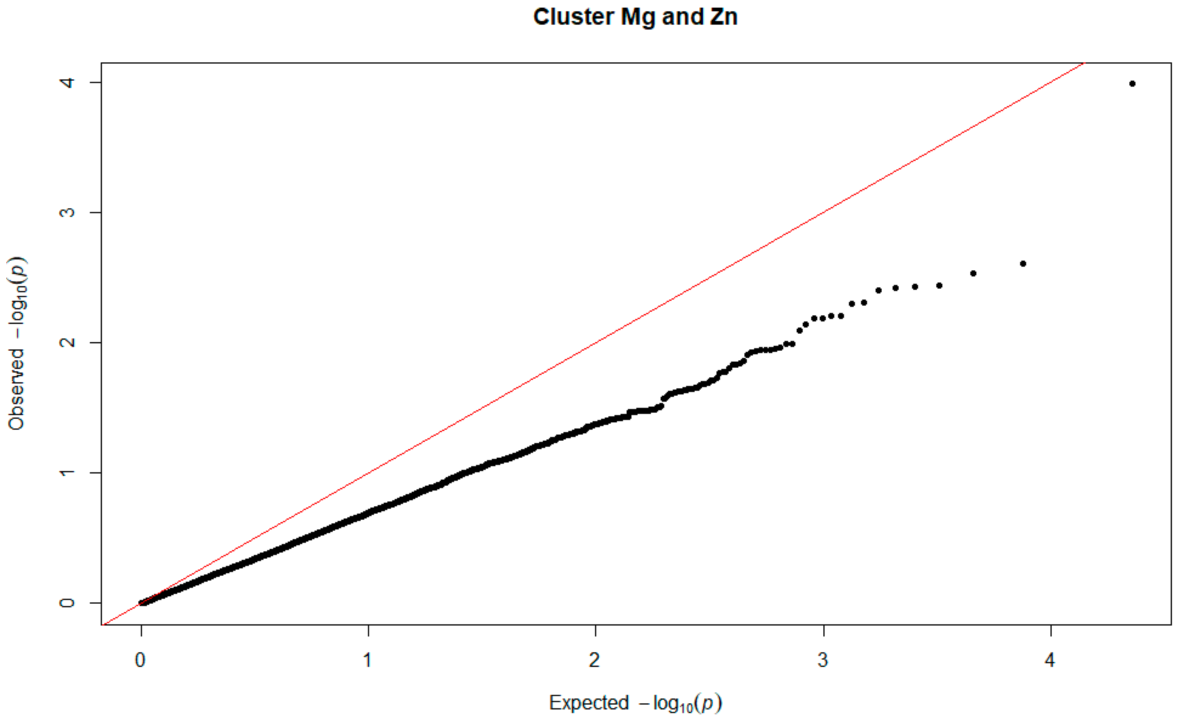

Supplementary Figure S19. Q-Q plot for a Mg and Zn cluster determined in multivariate analysis

.

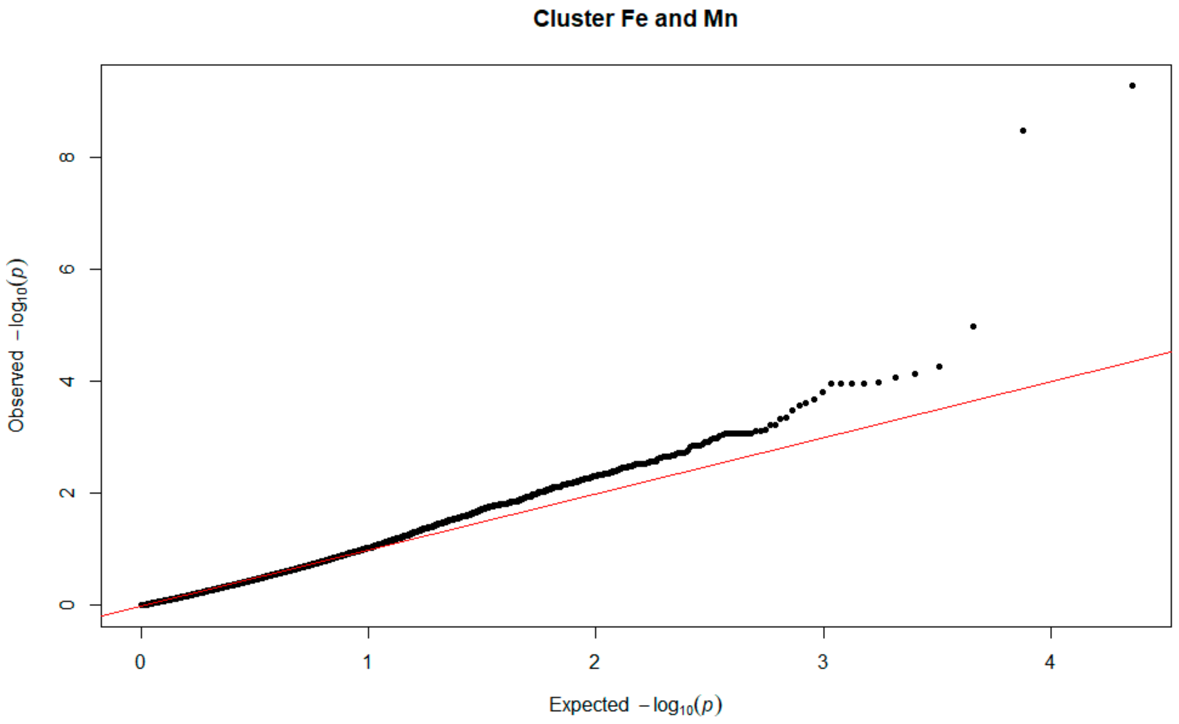

Supplementary Figure S20. Q-Q plot for a Fe and Mn cluster determined in multivariate analysis.

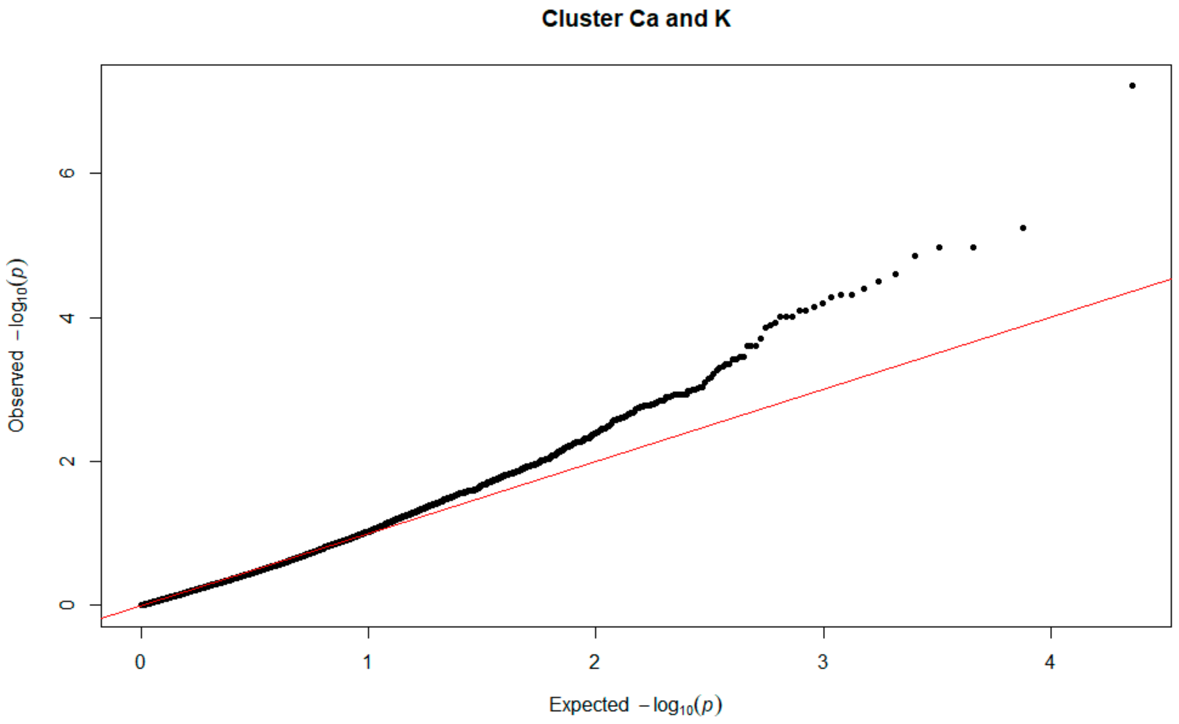

Supplementary Figure S21. Q-Q plot for a Ca and K cluster determined in multivariate analysis.

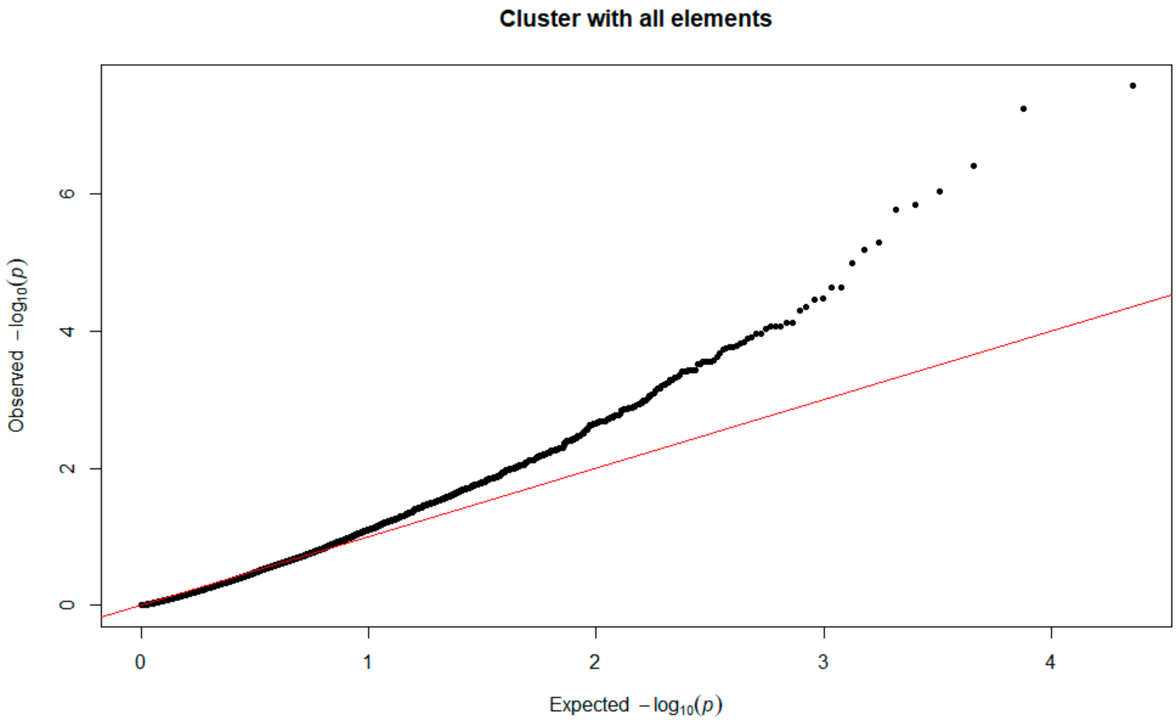

Supplementary Figure S22. Q-Q plot for all the elements considered jointly in multivariate analysis.
